# Extract of a Letter from Mr. Robert Chessher, Surgeon at Hinckley, in Leicestershire; Containing an Account of a Case of Luxation of the Os Humeri, in Which the Reduction of the Bone Was Facilitated by Inducing Sickness and Faintness by Means of Emetic Tartar

**Published:** 1787

**Authors:** 

**Affiliations:** Physician in London


XIII. *Extract of a Letter from Mr.* Robert Chefsher, *Surgeon at Hinckley, in Leiceſterſhire; containing an Account of a Caſe of Luxation of the Os Humeri, in which the Reduction of the Bone was facilitated by inducing Sickneſs and Faintneſs by Means of Emetic Tartar. Communicated to Dr.* Simmons *by Dr.* Denman, *Phyſician in London.*

SOME time ago I was called to a very robuſt man, whoſe ſhoulder was diſlocated, the head of the bone being ſlipped pretty far under the pectoral muſcle. Many fruitleſs attempts had been made to reduce it. I was at that time ill, and not feeling able to exert myſelf in ſuch a manner as to have a chance of overcoming the difficulty, I gave him, while he he was fitting on the chair, a folution of emetic tartar in mint water. In a fhort fpace of time it was repeated, and, after the third dofe, he became fick, and fo faint, that he could fcarcely fupport himfelf in the chair. During this ftate of faintnefs, I directed a moderate extenfion to be made, and then guiding the head of the bone, it immediately, and with great eafe, was returned into the focket *.

* In the Philofophical Tranfactions, Vol. LI. the reader will find a cafe of luxated thigh bone, by Mr. Yonge, Surgeon at Plymouth, in which "the mufcles were fo much weakened "by the patient's being confined to his bed, and wafted by "frequently repeated purges, that they very eafily gave way," and the reduction was effected on the twenty-fifth day, after having been in vain attempted by the ufual mode of extenfion immediately after the accident. The event of that cafe led Mr. Yonge very ingenioufly to fuggeft, whether "the giving "ftrong purgatives, and frequently repeating them, fo as to "render the mufcles of ftrong mufcular fubjects more lax and "weak, might not be employed with advantage in luxations of "the os humeri;" and the cafe related by Mr. Chefsher fhews how well this idea was founded. — EDITOR.

XIV. *Obfer-*

